# Effect of Increased Water Intake on Urinary DNA Adduct Levels and Mutagenicity in Smokers: A Randomized Study

**DOI:** 10.1155/2015/478150

**Published:** 2015-08-18

**Authors:** Inmaculada Buendia Jimenez, Pascaline Richardot, Pascaline Picard, Eve M. Lepicard, Michel De Meo, Glenn Talaska

**Affiliations:** ^1^Danone Research, avenue de la Vauve, 91120 Palaiseau, France; ^2^Aixial Pharma, 92300 Levallois-Perret, France; ^3^Institute of European Expertise in Physiology, 21 rue Leblanc, 75015 Paris, France; ^4^IMBE, UMR CNRS 7263, IRD 237, Faculté de Pharmacie, Aix-Marseille Université, 13331 Marseille, France; ^5^Department of Environmental Health, College of Medicine, University of Cincinnati, 38 Woodsdale Avenue, Cincinnati, OH 45216, USA

## Abstract

The association between fluid intake and bladder cancer risk remains controversial. Very little is known about to which extent the amount of water intake influences the action of excreting toxics upon the urinary system. This proof of concept trial investigates the effect of water intake on mutagenesis in smokers, a high risk population for bladder cancer.* Methods*. Monocentric randomized controlled trial.* Inclusion Criteria.* Male subjects aged 2045–45 y/o, smokers, and small drinkers (24-hour urinary volume <1 L and osmolality >700 mOsmol/kg).* Outcomes.* 4-ABP DNA adducts formation in exfoliated bladder cells in 24-hour urine collection and urinary mutagenicity in 24-hour urine.* Test Group.* Subjects consumed 1.5 L daily of the study product (EVIAN) on top of their usual water intake for 50 days.* Control Group.* Subjects continued their usual lifestyle habits.* Results.* 65 subjects were randomized. Mean age was 30 y/o and mean cigarettes per day were 20. A slight decrease in adducts formation was observed between baseline and last visit but no statistically significant difference was demonstrated between the groups. Urinary mutagenicity significantly decreased. The study shows that increasing water intake decreases urinary mutagenicity. It is not confirmed by urinary adducts formation. Further research would be necessary.

## 1. Introduction

Tobacco smoking and occupational exposure to chemicals are leading causes of urinary bladder cancer [1]. This is thought to be largely due to exposure to aromatic amines, which, when activated, can react with DNA to form DNA adducts that may induce mutations in key cancer-related genes [1]. Metabolites of aromatic amines are mainly excreted in the urine [2–4], and bladder biopsies and exfoliated urothelial cells from exposed populations contain increased levels of DNA adducts specifically associated with bladder cancer, benzidine and 4-aminobiphenyl (4-ABP) [5, 6]. Since smokers and workers exposed to polycyclic aromatic hydrocarbons (PAH) also excrete increased amounts of mutagens into their urine, it is possible that PAH also contribute to the increased risk of urinary bladder cancer. Indeed, we have recently reported that PAH exposure in coke oven workers is associated with increased levels of DNA adducts that are correlated with levels of urinary 1-hydroxypyrene in the absence of aromatic amine exposure [7]. The mutagenic profile of smokers urine suggests that both aromatic amines and PAH materials are excreted at increased levels. Increasing fluid intake could potentially reduce the impact of urinary bladder carcinogen exposure, either through simple dilution and/or by decreasing the time the carcinogen spends in the urinary bladder because of increased micturition frequency. In either case one might expect that an increase in water intake would induce lower levels of urinary mutagens, DNA adducts in the urinary bladder, and, perhaps, the risk of urinary bladder cancer.

Results from epidemiological studies investigating the association between fluid intake and bladder cancer have been inconsistent. Recently, Ros et al. reported no association between total fluid intake and bladder cancer using data from the European Prospective Investigation into Cancer and Nutrition cohort study [8]. On the other hand, in two large case-control studies, Michaud et al. found an inverse association between total fluid and plain water intake and the risk of bladder cancer, although no such relationship was found for other beverages [9, 10]. Jiang et al. also reported an association between daily water intake and a slight decrease in bladder cancer risk as well as an inverse relationship between urination frequency and bladder cancer risk for subjects who urinated at least six times per day [11]. Furthermore, the risk of bladder cancer is inversely related to nighttime voiding frequency [12], and urination frequency appears to be one of the four main factors contributing to interindividual differences in DNA binding by 4-ABP in human bladder [13]. Finally, experiments in dogs indicate that exposure to N-hydroxy-4-ABP and 4-ABP-DNA adduct formation in the bladder are inversely correlated with urination frequency [14].

Consequently, further investigation of the ability of increased water intake to reduce the risk of bladder cancer and exposure to aromatic amines is warranted. Our hypothesis was that increasing water intake will reduce the exposure of urothelial cells to mutagens in the urine. First, increasing water intake leads to an increased urine output. This means that urine is diluted, that is, less concentrated in toxic substances. Second, increased urination frequency decreases the duration of urothelial cell exposure to toxic substances. Thus, increased water intake should lead to decreased exposure concentration for less time and reduce the contact time of the mutagens with the target cells. To the best of our knowledge, this hypothesis has not been investigated in humans previously. Here, we report the results of a proof-of-concept, randomized, controlled study on the effects of increased bottled water intake on urinary DNA adduct levels and mutagenicity in moderate to heavy smokers.

## 2. Materials and Methods

### 2.1. Study Design

This was a single-center, randomized, controlled, open-label, two-parallel group study in healthy male low water consumers who smoke (ClinicalTrials.gov no. NCT01583387). The study was conducted at Centre CAP (Montpellier, France) between February 21 and July 4, 2011. The primary objectives of this study were to assess the effect of increased water intake on the mutagenicity of smokers' urine and subsequent formation of 4-ABP and BPDE-DNA adducts in exfoliated bladder cells in 24-hour urine.

### 2.2. Ethics

The study was approved by the independent ethics committee of Sud Mediterranée III, France, and the Agence Nationale de Sécurité du Médicament et des Produits de Santé, France, and it was carried out in accordance with the Declaration of Helsinki and the International Conference on Harmonization Guidelines for Good Clinical Practice. All subjects gave their written informed consent before inclusion in the study.

### 2.3. Subjects

Male smokers 20 to 45 years of age were recruited for this study. Potential subjects were screened about their smoking habits by telephone. To be included in the study, they had to have a moderate or high level of dependence on cigarette smoking as indicated by a score ≥5 on the Fagerström Test for Nicotine Dependence [15] and had to smoke ≥15 cigarettes/day for the last 2 years. Those eligible had an initial visit with the study investigator, where they were given a diary in which they recorded dietary and smoking habits for 2 weeks. To be included and randomized, subjects had to drink ≤1 L of fluid and ≤500 mL water per day, have a urine osmolality >700 mOsmol/kg, have a body mass index of 18–27 kg/m^2^, had to eat 3 meals per day, and had to be covered by the French national health insurance system. Subjects were excluded if they had a history of metabolic disease or acute or chronic gastrointestinal disease except appendectomy; were diagnosed with urinary tract, lung, or respiratory disease; regularly consumed more than three units (12 g) of alcohol per day; or were taking or addicted to drugs (e.g., cannabis, opioids, or amphetamines). Subjects were also excluded if they handled paints, dry cleaning chemicals, dyes, pesticides, aluminum, or asphalt; ate grilled foods more than three times per week; were vegetarian; participated in intense physical activity; could not or were not willing to increase their fluid intake by 1.5 L per day; were planning to stop or reduce their smoking or change brand of cigarettes during the study; or had evidence or a history of disease, were receiving treatment, or had any other situation that, in the investigator's opinion, could affect the study parameters.

### 2.4. Study Conduct

Eligible subjects were randomized 1 : 1 to the test or control group. The randomization list was generated using SAS version 9.2 (SAS Institute, Cary, NC), and subjects were assigned to groups sequentially. Subjects assigned to the test group had to consume three 500 mL bottles daily of natural mineral water (Evian, Danone, Paris, France) in addition to their usual water intake for 50 consecutive days. Bottles were opened at the beginning of every meal (breakfast, lunch, and dinner) and had to be fully consumed before the next meal. Subjects in the control group continued their normal fluid consumption.

At screening, investigators recorded physical characteristics, demographics, smoking behavior, alcohol consumption behavior, medical history, and concomitant medication. In addition, smoking habits were assessed at randomization and study end using the Fagerström Test for Nicotine Dependence [15].

At baseline and at the end of the study, subjects were asked to collect 24-hour urine. They were trained by the study nurses to obtain a complete collection and to store the samples under appropriate conditions (approximately 4°C). The first urine on day 1 was excluded. If one urination was missing, the subject was asked to make a new 24-hour urine collection. Blood samples (18 mL) were collected from subjects. Subjects were asked to complete an online food and fluid questionnaire for 3 days before both visits. Information in online diaries included meal type (breakfast, lunch, dinner, or snack), time, location, composition, and quantity of each component; type of fluid intake and time and quantity of each consumption; and number of urinations per day. Subjects also completed the fluid intake diary once per week during the intervention period. Finally, the test group was also provided with a printed diary in which they recorded their daily consumption of each bottle of water administered for the study.

### 2.5. Urine Analysis

The 24-hour urine was collected in a specific (sterile container of 2.7 L with screw cap, without preservative) container during 24 h and stored at about +4°C to ensure the stability of analysed parameters. Then for DNA adduct analysis, a volume of a 50% glycerol solution was added to the containers (one volume of glycerol solution was added to four volumes of urine) to reduce cell lysis during the freezing-thawing of the samples. Urinary mutagenicity was assessed with the* Salmonella*-microsome assay using the strain* Salmonella typhimurium* YG1041 in the presence of the metabolic fraction S9 Mix [16]. Exfoliated bladder cells were isolated and 4-ABP-DNA and BPDE-DNA adducts in exfoliated bladder cells were measured as previously described [16, 17]. The values for each individual were the mean of at least two independent replicate samples. For each DNA adduct, results are expressed as the number of adducts per 10^8^ unadducted nucleotides. Total volume of urine was calculated as urine weight (g) ÷ urine specific gravity (g/L).

### 2.6. Safety

At each visit, subjects were asked if they had experienced any adverse events (AE) since the last visit. AEs were defined as any unwanted effect whether or not related to the study product. Abnormal laboratory results were not considered AEs except when indicative of disease or organ toxicity. For each AE, investigators recorded the severity (mild, moderate, and severe), relationship to the study product (unrelated or unlikely, possibly, probably, or definitely related), and treatments for each event. Severe adverse events were defined as any AE that resulted in death; was life-threatening; resulted in disability, permanent incapacity, hospitalization, or prolonged hospitalization; or was medically important in the investigator's opinion.

### 2.7. Determination of Sample Size

Due to a lack of information on clinically relevant effects, the sample size was arbitrarily set at 30 subjects per group. This sample size was estimated to provide 35% precision for determination of the primary outcome measure (4-ABP-DNA level) and the minimum needed to perform analysis of variance (ANOVA). Considering an estimated 10% dropout of enrolled subjects, a total of 66 subjects were planned.

### 2.8. Statistical Analysis

Statistical analysis was performed using SAS version 9.2. Safety, demographics, and baseline characteristics were assessed in the full analysis set, which included all subjects randomized. Primary and secondary endpoints were assessed in all subjects completing the study according to protocol as well as in all subjects randomized and for whom data were available. For continuous outcomes, the main model was a generalized linear model on the raw change of the value with the study group as fixed factor, value at baseline, and number of cigarettes consumption at baseline as covariate. All adduct measures were log-transformed to provide normally distributed data. Before being log-transformed, the measures equal to 0 were replaced by 0.1 (limit of detection divided by two). The underlying assumptions of normality of residuals for analysis of variance model were checked by using skewness and kurtosis statistics. A distribution was considered as approximately normal if the values of skewness and kurtosis fell within the interval of −1.5 to 1.5. In case of nonnormality, a nonparametric analysis (Wilcoxon test) was performed. Differences were considered statistically significant at *p* < 0.05.

## 3. Results

### 3.1. Safety

No treatment-related adverse events were reported, and no clinically significant abnormalities and no serious adverse events were reported. Biological parameters, urinalysis dipstick results, and vital signs remained stable during the study (data not shown).

### 3.2. Demographics and Baseline Characteristics

A total of 118 male smokers were contacted and 110 were enrolled. Of these, 65 were eligible for randomization after the 2-week screening period. Of these, 33 were randomized into the test group and 32 into the control group. In both groups, the subjects were 20 to 45 years of age and baseline characteristics and tobacco and drinking habits were similar (Table 1). All subjects smoked blond tobacco, most smoked exclusively cigarettes, and most smoked filtered cigarettes.

Two subjects from the test group and one subject from the control group voluntarily withdrew from the study before its completion on day 50. Thus, 31 subjects in each group completed the study.

### 3.3. Fluid Intake and Water Intake Compliance

According to data collected from online diaries, total fluid intake at baseline was 600.8 ± 173.8 mL for the test group and 602.3 ± 165.2 for the control group. Total fluid intake increased on average (adjusted mean ± standard error) to 2127.2 ± 243.5 mL in the test group but changed little (602.0 ± 153.1 mL) in the control group. Mean water intake (± standard deviation (SD)) in the test group increased from 247.2 ± 104.1 mL at baseline to 1612.3 ± 131.6 mL at study end. Water intake in the control group remained relatively stable, starting at 229.1 ± 98.9 mL at baseline and finishing at 257.5 ± 100.0 mL at study end. Subjects in the test group consumed between 98% and 108% (median, 100%) of the additional 1.5 L of water that they were instructed to drink.

In the test group, the urine volume increased from 0.789 ± 0.164 L to 2.112 ± 0.607 L at the end of the study. Accordingly, the number of urinations per day increased from 3.7 ± 1.6 to 6.7 ± 2.3. By contrast, in the control group, the change in urine volume from baseline was −0.04 ± 0.07 L and in the number of urinations/day was −0.2 ± 0.9. Blood osmolality, which reflects intracellular osmolality, decreased significantly in the test group as compared to the control group, confirming the dilution effect of increased water intake (Table 2). Consistently, urine osmolality and urine specific gravity also significantly decreased (Table 3). These results indicate good compliance of water intake by the test group and no change in water intake by the control group.

### 3.4. Smoking Habits

The mean number of cigarettes consumed/day (±SD) remained stable between baseline and study end in both the test group (from 20.5 ± 5.4 to 20.7 ± 4.8) and the control group (20.3 ± 4.0 to 21.3 ± 3.8), with no difference in the change (0.2 ± 3.6 for the test group versus 0.8 ± 3.2 for the control group; *p* = 0.121). In addition, mean Fagerström scores (±SD) were similar and changed little between baseline and study end in the test group (from 7.2 ± 1.4 to 7.4 ± 1.3) and in the control group (from 7.2 ± 1.3 to 7.4 ± 1.1).

### 3.5. Nutrient Intake

Changes in nutrient consumption (total energy and grams carbohydrate, protein, fat, fiber, and sodium) between baseline and study end were not significantly different between the test and control groups (data not shown).

### 3.6. Urinary Mutagenicity

Mutagenic activity of the urine in vitro was decreased significantly in the test group from 206 revertants/mL to 101 revertants/mL by the end of the study. This indicates a mean net loss of ±SD of 114.19 ± 119.49 revertants/mL (53.6% decrease) in the test group, whereas in the control group, mutagenic activity changed little (140.9 revertants/mL at initial assay and 153 revertants/mL at completion). The decrease in mutagenic activity was significantly greater in the test group (*p* < 0.001).

These data taken together indicate that although the smoking habits and excretion of total mutagens in the urine did not change in the study population, the increased urine output induced a concomitant dilution of mutagens excreted in urine. In addition, since urine output increased in the test group, the urinary frequency and therefore the time urothelial cells were exposed to mutagens decreased. Thus, the bladder cells were exposed to significantly more dilute urine for a shorter amount of time between voids.

### 3.7. DNA Adduct Levels in Exfoliated Urothelial Cells

In subjects completing the study according to protocol, mean 4-ABP-DNA and BPDE-DNA adduct levels were between 0.4 and 0.8 per 10^8^ nonadducted nucleotides (Table 4). The levels of these adducts decreased slightly (<0.2 per 10^8^ nonadducted nucleotides) between baseline and study end but with no significant differences between groups. Adduct levels at both baseline and study end were highly variable, as indicated by large SDs.

Results were similar when assessed in the full set of all randomized subjects (data not shown). No correlation was found between urinary mutagenicity and 4-ABP-DNA or BPDE-DNA adducts formation (data not shown).

## 4. Discussion

Several hypotheses have been proposed for the mechanism by which fluid intake may decrease the risk of bladder cancer, although the “urogenous contact hypothesis” is the most commonly considered. Braver et al. observed that the incidence of bladder cancer differs in urban and rural working populations and that this difference correlates with urine concentration and less frequent micturition [17]. Accordingly, prolonged contact of carcinogens in the urine with the urothelium was proposed to increase bladder cancer risk. According to this model, higher fluid intake, which should result in a higher volume and frequency of urination, would reduce the contact time and limit the effects of carcinogens on bladder tissue [12]. Mechanistic studies in animals support this hypothesis, but data from humans are lacking [14].

We designed a controlled proof-of-concept trial to assess the effect of increased water intake on DNA adduct formation and mutagenicity in the urothelial cells from smokers. For this study, we recruited 64 smokers with low fluid intake (<1 L/day). Recruiting subjects with such a low total fluid intake was easily achieved, in agreement with Vergne et al. who reported that 21% of the French adult population has a fluid intake lower than 1 L/day [18].

This study did not find a difference in the levels of the tested adducts in the urine of subjects who had an increase of water intake compared to the low water intake control group. We suspect that the lack of a significant effect on DNA adducts was principally due to high interindividual variability in DNA adduct levels, assay variability and the relatively small sample size, and the relatively short treatment period, which may not have allowed full expression of the differences to become manifest. In fact, previous studies have noted high interindividual differences in adduct levels. For example, DNA binding by 4-ABP is estimated to vary between individuals by as much as one millionfold between the most and least susceptible individuals [13]. The ability to detect differences in DNA adducts could also have been reduced by insufficient turnover of the urothelium during the 50 days of this study; the lifetime of the urothelium has been estimated to be 50 to 200 days [19], and a turnover rate at the high end of this range would have reduced the ability to detect differences. Alternatively, a larger sample size will be necessary to detect significant differences.

Urinary mutagenicity was also assessed in our study as a complementary test to evaluate the mutagenic potential of the collected urine. Mutagenic activity was significantly lower in the test group than in the control group. This result seems to be in line with the urogenous contact theory [18]: abundant water intake, and consequently diluted urine, would decrease the time of contact between the carcinogens in urine and DNA and lead to a decrease in the number of mutations. However, the mutagenicity test employed prokaryotic cells, which might not necessarily be predictive of mutagenicity in mammalian cells.

DNA adducts in the exfoliated urothelium and urinary mutagenicity integrate exposure to mutagens over very different amounts of time. The concentration of urinary mutagens is essentially a measure of short-term exposure and is expected to decrease rapidly if the amount of mutagen remains the same but the urine volume increased. In contrast, DNA adduct levels in exfoliated urothelial cells integrate exposure over the lifespan of the urothelium, which has been variously estimated to be 50 to 200 days [20]. Therefore, even a 50% reduction in exposure (as indicated by urine mutagenicity) would be only slowly translated into reduced levels of DNA adducts as the urothelial cells are exfoliated. Reduction in adduct levels would be slower if the lifespan of the urothelium was greater than 50 days. In support of this hypothesis, Henn et al. reported that the levels of urinary metabolites are only slightly and nonsignificantly lower in the wives of nonsmokers than in the wives of smokers but that DNA adduct levels were significantly lower in the wives of nonsmokers, suggesting that chronic higher exposure to mutagens in secondhand smoke eventually caused a significant increase in carcinogenic DNA adduct levels [20].

To date, most investigations on the effects of water intake on bladder cancer have been retrospective analyses of observational data [9–13]. These are susceptible to bias, and we wanted to use a strong methodological approach, so we designed a randomized, controlled, parallel-group study in which the eligibility criteria were narrowly defined and in which natural mineral water with a low mineral content was used. This reduced the influence of potential confounders, such as concomitant illnesses, tap water contaminants, and high mineral content. We also confirmed that compliance with the study procedures was excellent and that dietary and smoking habits did not significantly differ between the groups.

## 5. Conclusion

As far as we know, this is the first time that the hypothesis that increased water intake significantly decreases urinary mutagenicity has been proven. Consequently, increasing water intake might be a low cost and safe approach to eventually reduce risk of mutagenesis in exposed populations. However, this hypothesis needs to be confirmed with long term and statistically well-powered clinical studies. Our investigation yielded important data that will be useful for the design of these future studies.

## Figures and Tables

**Table 1:** Subject demographics and baseline characteristics.

Characteristic	Test group (*N* = 32)	Control group (*N* = 32)
Age (y)		
Mean ± SD	28.5 ± 7.4	30.6 ± 6.8
Range	20–45	20–45
Weight (kg)		
Mean ± SD	71.88 ± 10.55	74.52 ± 7.78
Range	54.0–94.0	59.5–91.0
Body mass index (km/m^2^)		
Mean ± SD	22.46 ± 3.03	23.51 ± 2.25
Range	18.1–27.0	18.6–26.9
Type of tobacco		
Blond	32 (100%)	32 (100%)
Filter use		
Yes	30 (93.8%)	28 (87.5%)
No	2 (6.3%)	4 (12.5%)
Tar/cigarette		
<3 mg	0 (0.0%)	0 (0.0%)
3–6 mg	1 (3.1%)	1 (3.1%)
7–10 mg	26 (81.3%)	29 (90.6%)
>10 mg	2 (6.3%)	0 (0.0%)
Unknown	3 (9.4%)	2 (6.3%)
Smoking in addition to cigarettes		
None	31 (96.9%)	28 (87.5%)
Cigarillos	0 (0.0%)	1 (3.1%)
Water pipes	1 (3.1%)	2 (6.3%)
Other	0 (0.0%)	1 (3.1%)
Cigarette smoking/day by others in the household		
None	22 (68.8%)	16 (50.0%)
≤10	6 (27.3%)	2 (12.5%)
11–20	8 (36.4%)	9 (56.3%)
21–30	2 (9.1%)	4 (25.0%)
>30	6 (27.3%)	1 (6.3%)
Fagerström score		
Mean ± SD	6.9 ± 1.4	7.0 ± 1.3
Range	5–10	5–9
Cigarettes smoked/day		
Mean ± SD	19.9 ± 6.2	20.8 ± 6.1
Range	15–40	15–40
Duration of smoking (y)		
Mean ± SD	11.3 ± 6.8	14.6 ± 6.7
Range	3–28	4–28
Alcohol consumption (units/day)		
<2	26 (81.3%)	27 (84.4%)
2 or 3	6 (18.8%)	5 (15.6%)
>3	0 (0.0%)	0 (0.0%)

Values are shown for all subjects randomized and for whom data were available. Note that no data were available for one subject randomized to the test group. SD, standard deviation.

**Table 2:** Blood chemistry changes after intervention in test group versus control group.

Measure	Test	Control	All	*P* value
	*n* (mean ± SD)	*n* (mean ± SD)	*n* (mean ± SD)	
Osmolality (mOsm/kg)				
Baseline	32 (300.3 ± 4.7)	32 (298.8 ± 6.5)	64 (299.5 ± 5.6)	
Study end	31 (297.0 ± 2.8)	31 (300.1 ± 4.1)	62 (298.5 ± 3.8)	
Absolute raw change	31 (−3.4 ± 5.4)	31 (1.3 ± 7.7)	62 (−1.0 ± 7.0)	
Adjusted mean change (SE)	−2.6 ± 0.6	0.5 ± 0.6		0.001

Values are shown for all subjects randomized and for whom data were available. SD, standard deviation; SE, standard error of the mean.

*P* value determined by analysis of covariance, adjusted for baseline values (parameter and cigarettes consumption).

**Table 3:** Urinary chemistry changes after intervention in test group versus control group.

Measure	Test	Control	All	*P* value
	*n* (mean ± SD)	*n* (mean ± SD)	*n* (mean ± SD)	
*Excretion *				
Osmolality (mOsm/kg)				
Baseline	32 (910.7 ± 105.3)	32 (908.2 ± 113.7)	64 (909.5 ± 108.7)	
Study end	31 (410.4 ± 130.6)	31 (827.1 ± 224.2)	62 (618.7 ± 277.9)	
Absolute raw change	31 (−502.1 ± 164.3)	31 (−84.8 ± 215.4)	62 (−293.5 ± 283.5)	
Adjusted mean change (SE)	−501.7 ± 32.5	−85.2 ± 32.5		<0.001
Urine specific gravity (g/mL)				
Baseline	32 (1.0246 ± 0.0033)	32 (1.0252 ± 0.0034)	64 (1.0249 ± 0.0033)	
Study end	31 (1.0115 ± 0.0037)	31 (1.0231 ± 0.0059)	62 (1.0173 ± 0.0076)	
Absolute raw change	31 (−0.0131 ± 0.0049)	31 (−0.0022 ± 0.0063)	62 (−0.0077 ± 0.0079)	
Adjusted mean change (SE)	−0.013 ± 0.0009	−0.0019 ± 0.0009		<0.001

Values are shown for all subjects randomized and for whom data were available. SD, standard deviation; SE, standard error of the mean.

*P* value determined by analysis of covariance, adjusted for baseline values (parameter and cigarettes consumption).

**Table 4:** Adducts and mutagenicity.

Measure	Test	Control	All	*P* value
	*n* (mean ± SD)	*n* (mean ± SD)	*n* (mean ± SD)	
4-ABP-DNA log (adducts/10^8^ DNA unadducted nucleotides)				
Baseline	32 (0.545 ± 0.762)	32 (0.608 ± 0.834)	64 (0.577 ± 0.793)	
Study end	31 (0.502 ± 0.705)	31 (0.528 ± 0.696)	62 (0.515 ± 0.695)	
Absolute raw change	31 (−0.056 ± 1.087)	31 (−0.054 ± 0.930)	62 (−0.055 ± 1.003)	
Adjusted mean change (SE)	−0.068 ± 0.127	−0.042 ± 0.127		0.889^b^
BPDE-DNA log (adducts/10^8^ DNA unadducted nucleotides)				
Baseline	32 (0.551 ± 0.806)	32 (0.396 ± 0.948)	64 (0.473 ± 0.876)	
Study end	31 (0.378 ± 0.781)	31 (0.414 ± 0.825)	62 (0.396 ± 0.797)	
Absolute raw change	31 (−0.222 ± 1.017)	31 (0.037 ± 1.097)	62 (−0.093 ± 1.057)	
Adjusted mean change (SE)	−0.131 (0.144)	−0.054 (0.144)		0.708^b^
Mutagenic activity (revertants/mL)				
Baseline	32 (205.80 ± 138.82)	31 (140.89 ± 90.60)	63 (173.86 ± 121.15)	
Study end	30 (100.51 ± 53.67)	29 (152.97 ± 104.95)	59 (126.29 ± 86.35)	
Absolute raw change	30 (−108.53 ± 121.45)	28 (1.11 ± 61.46)	58 (−55.60 ± 111.12)	
Median [Q1; Q3] for raw change	−80.70 [−184.00; −24.10]	2.40 [−27.75; 32.70]		<0.001^a^

Values are shown for all subjects randomized and for whom data were available. SD, standard deviation; SE, standard error of the mean.

^a^ *P* value determined by Wilcoxon Rank-Sum test.

^b^ *P* value determined by analysis of covariance, adjusted for baseline values (parameter and cigarettes consumption).

## Abbreviations

PAH: Polycyclic aromatic hydrocarbons
4-ABP: Benzidine and 4-aminobiphenyl
BPDE-DNA: Benzo(a)pyrene diol epoxide
AE: Adverse events
ANOVA: Analysis of variance
SD: Standard deviation.
